# Clinical evaluation of open flap debridement with or without Emdogain® in treating intrabony defects

**DOI:** 10.6026/973206300220430

**Published:** 2026-01-31

**Authors:** Simrita Singh, Vandana Saranga, Sahib Tej Singh, Vidhuta Sareen, Simranjit Kaur, Sakshi Verma

**Affiliations:** 1 Department of Periodontology, Sri Guru Ram Das Institute of Dental Sciences and Research, Amritsar, Punjab, India

**Keywords:** Open flap debridement, Enamel matrix derivative (EMD), periodontal intrabony defects, Regenerative techniques, Clinical attachment gain

## Abstract

Periodontitis causes intrabony defects and while open flap debridement (OFD) with EMD and GTR shows promise, the comparative effectiveness of EMD in treating these defects remains underexplored. Open flap debridement (OFD) or access flap surgery is used in conjunction with biological factors such as enamel matrix derivative (EMD) and regenerative techniques involving guided tissue regeneration (GTR) in order to overcome these intrabony defects. In numerous clinical investigations, the application of EMD has demonstrated notable clinical improvements in bone fill clinical attachment gain and pocket depth reduction. Thus, the clinical effectiveness of open flap debridement with and without enamel matrix derivative (EMD) in treating periodontal intrabony defects, further exploring EMD's contribution to clinical outcomes such as bone fill, attachment gain and pocket depth reduction is reported.

## Background:

Periodontitis is a complicated disease with many underlying causes. Some of these causes are genetic, some are the result of epigenetic influences and some are modifiable due to patient behaviors, medication interactions, or environmental factors [1]. Formation of intrabony defect is one of the sequelae of periodontal disease process, which are alterations in the framework of alveolar bone [2]. They are characterized by angular or vertical defects that form obliquely, creating a hollowed-out trough in the bone next to the root. Prevention and halting the disease process from progressed to advanced stages remain the primary objective of therapy provided. First clinical step involved in this is mechanical removal of supragingival and sub gingival biofilm [3]. Open flap debridement (OFD) or access flap surgery was amongst the earliest surgical treatments utilized to create the optimal biological conditions needed for periodontal regeneration as it improves visibility and efficacy of sub gingival debridement by gaining direct access to the root surface, root concavities and furcation in sites with residual pockets irrespective of the pattern of bone restoration [4]. But conventional OFD limits the regeneration of lost tissue due to down growth of junctional epithelium along the denuded root surface apically. Further, regenerative materials are required that will regenerate only desired cells and prevent further formation of junctional epithelium. In clinical trials assessing regenerative methods like guided tissue regeneration (GTR) and the application of biological components like enamel matrix derivative (EMD), OFD has historically been used as the control approach [5]. EMDs were first developed in 1997 and were considered to tend to regenerate periodontal tissues [6]. The beginning of acellular cementum starts with the temporary deposition of enamel matrix proteins onto the dentinal root surface. This method induces mesenchymal cells to replicate the processes that occur during the development of the nascent root and periodontal tissues using an extract of embryonic enamel matrix [7]. It has also been demonstrated that 90% of the proteins that make up the enamel matrix, known as amelogenins, promote the activation and growth of human periodontal ligament fibroblasts in addition to the proliferation of periodontal ligament cells [8]. Amelogenins contains proline residues which are in abundance and are believed to hinder the secondary structures like α-helix, β-sheet and coils and thus disorganizing the proteins. This helps amelogenin to produce supramolecular aggregates which are called nanospheres by self-assembling and creating an insoluble extracellular matrix that regulates the ultrastructural arrangement of the enamel crystallites as they mature [9]. The self-assembly mechanism is regulated by pH, temperature, strength and protein concentration. So, amelogenin is insoluble at normal temperatures and pH; therefore, it requires both high or low pH and low temperature. Various root conditioners like propylene glycol alginate (PGA), ethylene diamine tetraacetic acid (EDTA) with acidic nature provide favourable conditions for EMD to precipitate on the exposed root surface at the surgical site [10]. This active state for regeneration remains for days and weeks to promote the growth and function of regenerative cells. Enamelin, ameloblastin (also known as amelin or sheathlin), amelotin, apin and many proteinases are among the other proteins that are present in the enamel matrix that also favours the growth of periodontal tissues [11]. A number of characteristics include the release of fibroblast growth factor, the induction of cementum formation, the growth of periodontal ligament cells proliferating, the formation of alveolar bone, inhibition of epithelial cell growth, increased synthesis of growth transforming factor (TGF-β), the reduction in extracellular matrix metalloproteinases (MMP-1) concentrations and blocking of osteoclast maturation [12]. Research investigations have verified, the utilization of Enamel Matrix Derivative that leads to a substantial pocket depth reduction, clinical attachment gain and bone fill [13]. Moreover, demonstration approved EMD significantly influences angiogenic activity and, soft tissue regeneration during wound healing. Further controlled clinical research also demonstrated that, in comparison to OFD alone, OFD with EMD resulted in a three times larger defect fill [14]. Therefore, it is of interest to compare and evaluate open flap debridement with or without use of EMD for management of periodontal IBD.

## Methodology:

Patients having established periodontitis indicative of flap visited the Department of Periodontology at our research institution had 24 defect sites investigated in this randomized controlled clinical trial. The research study was initially submitted to the institutional ethical committee. After approval, patients were informed verbally as well as written consent taken before including them as part of trial. The study was conducted in agreement with principles embodied in the Helsinki Declaration of 1975, as revised in 2013.

## Inclusion criteria:

Patients indicative of flap surgery clinically diagnosed with Generalised Stage III Grade B periodontitis. Intrabony defects with a probing pocket depth (PPD) of ≥ 5mm, relative attachment level (RAL) of ≥ 5mm and Intrabony defects ≥3mm on radiographs. Patients showing interest and were willing to be a part of the trial signed performa of consent.

## Exclusion criteria:

Any systemic condition that might influence the results of trial, any periodontal therapy undertaken within past 6 months, any drug abuse and pregnant and lactating mothers were all excluded.

## Study design:

The participants who met the inclusion and exclusion criteria were assigned into two groups: Group A and Group B. In Group A, 12 defect sites were treated with open flap debridement while in Group B, 12 defect sites were treated with open flap debridement along with application of Emdogain®. The clinical parameters were gingival index, pocket probing depth and clinical attachment level whereas radiographic assessment of the intrabony defects were also recorded at baseline, 3 months and 6 months. The surgical procedure was started off with adequate local anesthesia (2% lignocaine and epinephrine), crevicular incisions were made on buccal aspect to the tip of the interdental papilla using a Bard-Parker handle and No. 15 blade. Periosteal elevator was used to raise flap bucally. Using Gracey curettes and 4R-4L Columbia universal curettes (Hu-Friedy), debridement was done to remove the degranulated tissue along with irrigation with normal saline. In Group A, only debridement of the tissue was done. In Group B, the root surfaces were treated with 24% Ethylene diamine tetra acetic acid (EDTA) for 2 minutes to remove the smear layer followed by rinsing with normal saline. Then Emdogain® was used to fill the defect sites. The flaps were approximated using 3-0 non-absorbable braided silk sutures. The flap surgery was secured with periodontal dressing (Coe-pak®) in both the groups. The data was collected, processed and finalized using SPSS version 17.0. At baseline, three and six months, paired t-tests were used within the group comparisons and unpaired t-tests used between the group comparisons to examine periodontal parameters for mean values, standard deviations and percentage changes. Follow-ups were done to reinforce oral hygiene instructions.

## Results:

**Figure 1:**
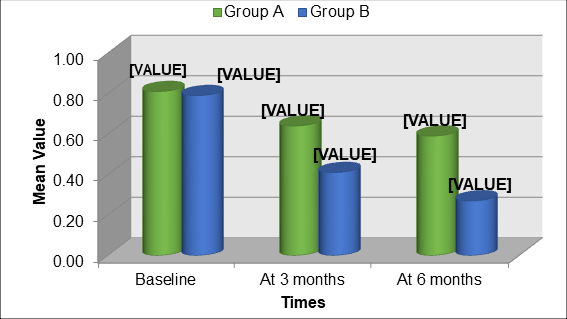
: Comparison of mean plaque score (pi) scores between group A (OFD) and B (OFD + EMDOGAIN®

**Figure 2:**
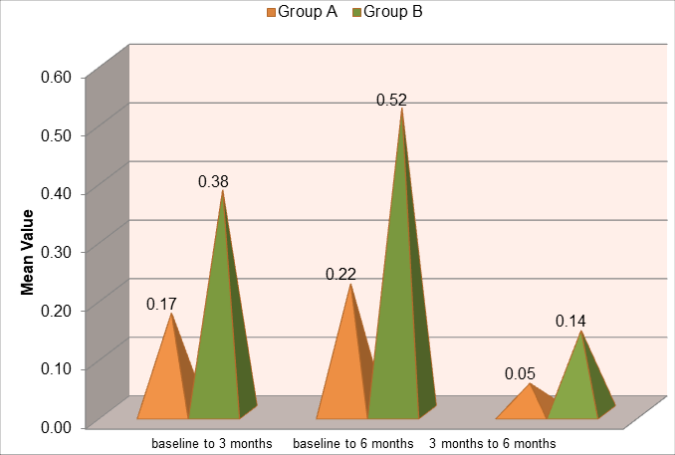
Comparison of mean change in plaque index (PI) scores between group a (OFD) and b (OFD + EMDOGAIN®

**Figure 3:**
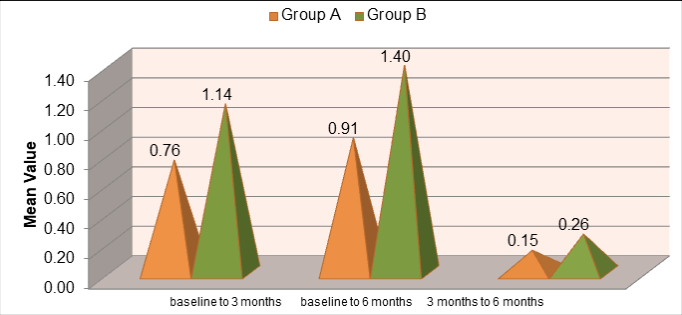
Comparison of mean change in gingival index (GI) scores in group a (OFD) and b (Ofd + EMDOGAIN®

The study involved 24 defect sites with Generalised Stage III Grade B periodontitis, with a mean age of 49.25 years (standard deviation 3.47) and comprised 54% females and 46% males. At baseline, Group A had a mean plaque index score of 0.81 ± 0.188. This score decreased to 0.64 ± 0.128 at 3 months and further to 0.59 ± 0.126 at 6 months. Conversely, Group B had a baseline plaque index score of 0.79 ± 0.179. This score reduced to 0.41 ± 0.123 at 3 months and to 0.27 ± 0.072 at 6 months (Figure 1). Mean plaque index scores were compared between groups A and B. The differences were determined to be statistically non-significant (p>0.05) at baseline, 3 and 6 months after surgery (Figure 2). Both groups demonstrated changes in plaque index three and six months post-operatively. The mean value of the gingival index score in group A was 1.46 ± 0.257, which reduced to 0.70 ± 0.094 at the end of 3 months and further reduced to 0.55 ± 0.099 at the end of 6 months. Whereas, the mean value of the gingival index score in group B was 1.60 ± 0.160, which reduced to 0.46 ± 0.107 at the end of 3 months and further reduced to 0.20± 0.095 at the end of 6 months. On comparison of mean values of gingival index scores between group A and B, the differences were found to be non-significant (p>0.05), at baseline and highly significant (p<0.001) at 3 and 6 months postoperatively (Figure 3). At 3 months post-treatment, Group A exhibited a mean reduction in probing pocket depth (PPD) of 6.81 ± 0.559 mm. By 6 months, the mean reduction increased to 5.85 ± 0.386 mm, with a change of 0.96 ± 1.374 mm observed between 3 and 6 months.

**Figure 4:**
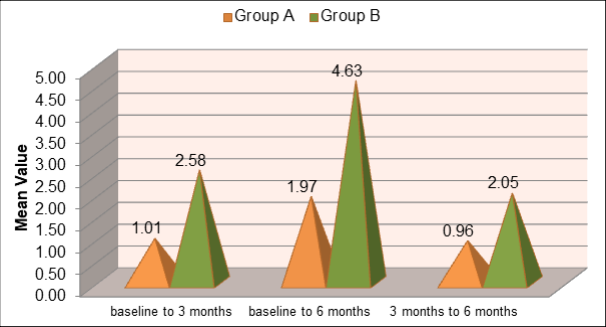
Comparison of mean change in probing pocket depth (PPD) in group A (OFD) AND group B (OFD + EMDOGAIN®

**Figure 5:**
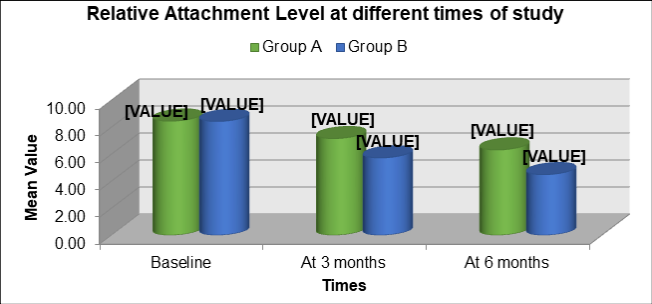
Comparison of mean clinical attachment level (CAL) between group A (OFD) and group B (OFD + EMDOGAIN®

**Figure 6:**
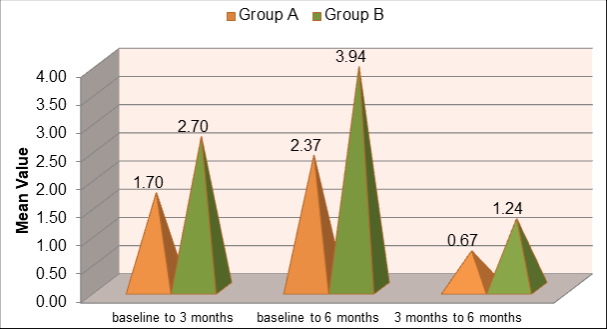
Comparison of Mean Change in Clinical Attachment Level (CAL) in group A (OFD) and group B (OFD + EMDOGAIN®

The decrease from baseline to 3 months and from baseline to 6 months was statistically highly significant, while the change between 3 and 6 months was also significant (Figure 4). In Group B, the mean PPD reduction at 3 months was 5.01 ± 0.370 mm, which increased to 2.96 ± 0.177 mm by 6 months. The reduction at 6 months was statistically significant, but the change between 3 and 6 months (2.05 ± 1.045 mm) was not important. The mean clinical attachment level (CAL) improved in both Group A (open flap debridement) and Group B (open flap debridement with Emdogain®) over the study period. However, Group B showed a greater gain in clinical attachment compared to Group A, indicating enhanced periodontal regeneration with the adjunctive use of enamel matrix derivative (Figure 5). The mean change in CAL from baseline to 3 and 6 months was observed in both groups. Group B demonstrated a significantly higher CAL gain than Group A, suggesting superior periodontal tissue regeneration when Emdogain® was used in addition to open flap debridement (Figure 6).

**Figure 7:**
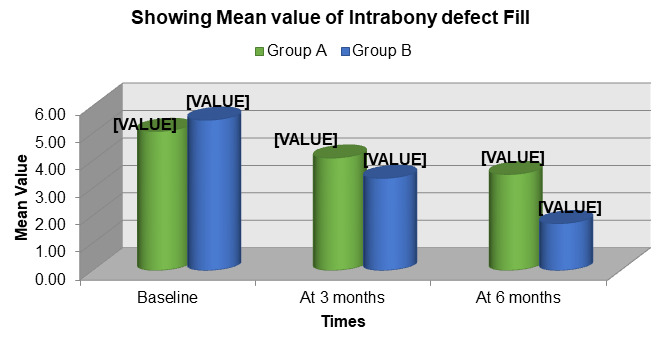
Comparison of mean intrabony defect (IBD) fill between group A (ofd) and group B (OFD + Emdogain®

**Figure 8:**
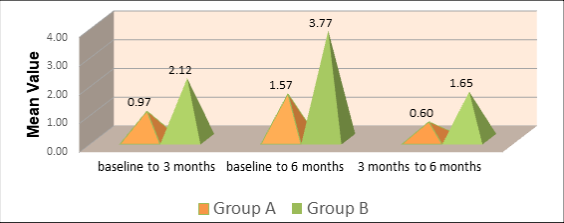
Comparison of mean change in intrabony defect (IBD) fill in group A (OFD) and group B (OFD + EMDOGAIN®

**Figure 9:**
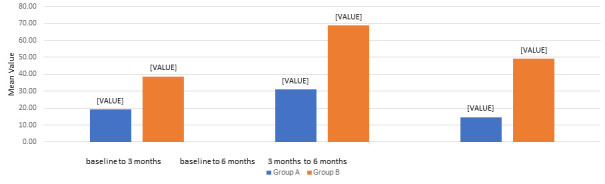
Comparison of mean intrabony defect (IBD) fill percentage (%) in group A (OFD) and group B (OFD + EMDOGAIN®

Radiographic assessment showed intrabony defect fill in both groups during follow-up. Group B exhibited a higher mean intrabony defect fill compared to Group A at both 3 and 6 months, indicating improved bone regeneration with the use of Emdogain® (Figure 7). The mean change in intrabony defect fill from baseline to follow-up was greater in Group B than in Group A. This suggests that enamel matrix derivative significantly enhanced bone formation and defect resolution compared to open flap debridement alone (Figure 8). The percentage of intrabony defect fill was higher in Group B compared to Group A at 6 months. The intergroup comparison showed a highly significant difference, confirming superior periodontal bone regeneration with Emdogain® compared to conventional surgical therapy (Figure 9).

## Discussion:

Plaque is a biofilm formed of salivary glycoproteins and extracellular polysaccharides which exclusively contains oral bacteria. Promoting appropriate self-performed oral hygiene and routine professional maintenance at regular intervals is a part of the prevention of bad oral hygiene habits [14]. Lavanchy *et al.* (1987) [15] showed that scaling and root planing had a significant positive effect in reducing gingival inflammation and healing of periodontal tissue due to reduction of bacterial plaque accumulation after 4 weeks of the treatment. There was plaque reduction in both the groups A and B and by three- and six-month, group B showed more plaque reduction as compared to group A. EMD is believed to have an impact on specific microorganisms with antibacterial effects and bacterial adherence impairment. Kadayif *et al.* (2025) [16] in a study showed that the reduction of Gingival Index following mechanical Non-Surgical Periodontal Therapy is an indication of favourable changes seen in the periodontium. The primary goal of periodontal therapy is to reduce the depth of the periodontal pocket since this makes the environment less favorable for the growth of anaerobic periopathogens. Cosgarea *et al.* [17] investigated in a study that a significant decrease in pocket depth post periodontal surgery was found, indicating better clinical results when the defect sites are surgically intervened, therefore emphasizing that surgical therapy is the cornerstone for periodontal pocket depth reduction. At three and six months, respectively, the comparison between the two groups was statistically significant, with group B showing a greater reduction in probing pocket depth and gain in clinical attachment level than group A. EMD adsorbs on decontamined root surfaces and alveolar bony defects and forms an insoluble scaffold complex. This complex promotes recolonization of periodontal cells, thus inducing periodontal regeneration. Tullio *et al.* [18] examined the clinical efficacy of EMD along with Simplified Papilla Preservation Flap (SPPF) in the treating of suprabony defects. After 12 months, EMD group showed improvements from baseline gain in Clinical attachment level, greater reduction in Pocket depth and less gingival recession. The study concludes that the gain in clinical healing is the result of epithelial and connective adhesion to the root surface. Periodontal diseases also affect the alveolar bone, leading to intrabony defects. So, the regeneration of infrabony defects remains the mainstay when deciding the success of the periodontal therapy outcomes. Intrabony defect fill was observed in both group A and B at 3 and 6 months from baseline. At six months, there was a highly significant difference in the amount of bone production between groups A and B, with group B having a higher percentage as compared to group A. Cheng *et al.* (2021) [19] showed in the study that EMD may have a strong, favourable impact on mesenchymal cell differentiation into mature bone cells by upregulating a number of crucial genes connected to bone. Galli *et al.* investigated that EMD did show increase the Osteoprotegrin/Receptor activator of nuclear factor kappa-β ligand (RANKL) ratio in osteoblast cultures and subsequently inhibit osteoclast formation in vitro, which may reduce local bone resorption and favour bone formation [20]. Enamel matrix derivative is a widely recognized biomaterial used for periodontal regeneration. It contains amelogenin taken from the Hertwig epithelial root sheath of developing porcine tooth buds. EMD influences the behaviour of different kinds of cells and effectively enhances the recovery of hard and soft tissues while reducing inflammation [21]. Despite additional benefits from the xenograft, Emdogain® showed promising results with less tissue morbidity and better patient compliance. To more precisely define and quantify the morphology of bone defects and investigate the long-term effects of Emdogain® when treating intrabony flaws, more research with larger samples and longer follow-up period is advised.

## Conclusion:

EMD has set a new trend for complete periodontal regeneration. It may lead to enhanced improvement in all the assessed parameters opposing OFD alone. Thus, use of EMD in periodontal therapy, exhibited significant improvement as compared to only periodontal therapy.
